# Nutrition literacy and socio-demographic determinants among Chinese women of childbearing age

**DOI:** 10.3389/fpubh.2026.1742415

**Published:** 2026-02-24

**Authors:** Jing-Jing Meng, Jun Chen, Li Pu, Yan Zhu, Yan Zuo, Fang Wang, Li Chang, Yi-Ying He, Jian-Jun Zhang, Zhi-Lan Bai, Si-Qin Sun, Jie Liu, Jia Shi

**Affiliations:** 1 Department of Gynaecology and Obstetrics, The Fourth Affiliated Hospital of Soochow University (Suzhou Dushu Lake Hospital), Suzhou, China; 2 Medical Services Management Department, Peking University People’s Hospital, Beijing, China; 3 Center for Health Statistics and Information, National Health Commission of People's Republic of China, Beijing, China; 4 Department of Gynecology and Obstetrics Nursing, West China Second University Hospital, Sichuan University, Chengdu, Sichuan, China; 5 West China School of Nursing, Sichuan University, Chengdu, Sichuan, China; 6 Key Laboratory of Birth Defects and Related Diseases of Women and Children (Sichuan University), Ministry of Education, Chengdu, China; 7 Zoucheng People’s Hospital, Zoucheng, China; 8 Hospital Management Center, Shulan Health Group, Hangzhou, China; 9 Neonatal Intensive Care Unit, Children’s Hospital of Soochow University, Suzhou, China; 10 Department of Gynecology and Obstetrics, West China Second University Hospital, Sichuan University, Chengdu, China; 11 Endocrinology Department, Zoucheng People’s Hospital, Zoucheng, China

**Keywords:** China, maternal health, nutrition literacy, reproductive health, socio-demographic determinants, women of childbearing age

## Abstract

**Background:**

Nutrition literacy is critical for women of childbearing age because it may shape dietary behaviors before and during pregnancy. Evidence on domain-specific nutrition literacy and associated factors in China remains limited.

**Methods:**

A cross-sectional online survey was conducted from August 20, 2024 to March 20, 2025, with dissemination supported by three tertiary hospitals in East, North, and Middle China. Women aged 18–49 years completed an adapted Chinese Nutrition Literacy Questionnaire (CNLQ). Domain and total scores were calculated using the original protocol and transformed to a 0–100 scale, with higher scores indicating higher nutrition literacy. Adequate nutrition literacy was defined as total score ≥80. Psychometric evaluation of the adapted instrument was performed in the full analytic sample. Univariable screening (*p* < 0.10) was used to select predictors for multivariable linear regression (total score) and multivariable logistic regression (adequacy).

**Results:**

Among 1,764 returned questionnaires, 1,391 were included after exclusions. Mean total nutrition literacy was 74.9 ± 12.8, and 33.4% (*n* = 465) met the adequacy threshold. Domain scores showed a gradient: functional 77.4 ± 13.4, interactive 75.1 ± 13.8, and critical 69.9 ± 14.2. In adjusted models, higher education, higher income, urban residence, and healthcare background were associated with higher total scores and higher odds of adequacy. Multimorbidity was associated with lower total score (adjusted *β* = −2.55) and lower odds of adequacy (adjusted OR = 0.62). Women aged 35–49 years had higher total scores (adjusted β = 1.67) and higher odds of adequacy (adjusted OR = 1.43) compared with those aged 18–24 years.

**Conclusion:**

Nutrition literacy among Chinese women of childbearing age is moderate, with critical literacy lowest. Socioeconomic factors, urban residence, healthcare background, and multimorbidity are key correlates, highlighting priorities for targeted interventions that strengthen critical appraisal skills and support women with multimorbidity.

## 1 Introduction

Nutrition literacy refers to a person’s ability to access, understand, critically evaluate and apply nutritional information for appropriate dietary decisions (1, 2). It has been positioned as an influential factor determining diet quality, health behaviors and clinical outcomes (3–5). In the wider health literacy domain, nutrition literacy is more than simply being informed of facts and knowledge but refers to interactive (i.e., one’s ability to effectively use information within social and personal settings) and critical nutrition (i.e., the ability to adapt nutritional information for complex social and clinical purposes). Over the past few years, studies have reported that higher nutrition literacy is linked to healthier dietary behaviors, a reduced risk of overweight and obesity, and more effective chronic disease self-management (6–8). Systamtic reviews have found that the relationship between dietary behavior and literacy level was consistent across populations (9–11).

Women of reproductive age play a pivotal part in the nutrition ecosystem. They not only maintain their own health but also determine the quality of nutritional intakes in their households. From clinical viewpoints,they also impact maternal-child outcomes. Chinese women aged 18–49 years face unique nutritional and reproductive challenges. Prior data reveal continued shortfalls of important micronutrients, increasing rates of overweight and obesity, and shifts to processed and high-sodium diets (12, 13). From a public health perspective, improved nutrition literacy within this key population could contribute to the achievement of wider national targets in China, as defined by the Healthy China 2030 Plan. But from a clinical/obstetric/gynecologic (OB/GYN) standpoint, the impact is perhaps even more direct. Maternal nutrition literacy can impacts gestational weight gain (GWG), status of micronutrients as well as prenatal care engagement and ultimately also maternal and fetal outcomes (14–16).

However, this leaves a significant missing link; there are little data on nutrition literacy, especially among non-pregnant women of reproductive age, particularly in the 18–49 year old group in either clinical or public health settings. Most previous research was conducted with general adult populations or was directed about nutrition knowledge, not literacy. For example, in one of the largest China-based studies conducted among 38,065 women aged 18–49 years, a mean nutrition knowledge score of 65.1 ± 11.8 and an awareness rate as low as 20.9% were found, which demonstrated the prevalence of inadequacies (17).

Although knowledge is important, literacy in the form of skills and behavior capacity (i.e., knowing how to decide and act) may be more applicable in clinical setting. From an OB/GYN viewpoint, women entering pregnancy, or planning a pregnancy or managing the aftermath of childbirth all can benefit from high nutrition literacy. In other word, adequate nutrition literacy can be helpful for healthier eating, appropriate weight gain, better supplement use, and improved pregnancy outcomes (18–20).

According to prior studies, nutrition literacy among Chinese women is relatively low, especially those in reproductive age and pregnancy. For example, a large-scale national surveys in 2021 showed that only about 20% of women aged 18–49 had adequate nutrition knowledge (17) In pregnant women, a large survey based study showed that only 3.9% of them score “excellent” level for nutrition literacy, and during the knowledge to actual healthy dietary behavior transition process most of them scored poorly (21). The nutrition literacy levels are significantly influenced by socio-demographic factors such as age, education, occupation and regions with lower indicators in rural areas and among women who have less education (21, 22). Interventions that combined online physical activity and dietary behavior change interventions have led to increased nutrition literacy, improved dietary quality, healthier gestational weight gain in urban pregnant women (18). Specialized nutrition literacy assessment tools validated among Chinese women have allowed for more focused monitoring and intervention (21). However, the demand for improved, culturally relevant nutrition education to translate knowledge into practice, and improve birth outcomes and the family’s health status is still pressing.

Therefore, continued monitoring of nutrition literacy among women of childbearing age in China is necessary to understand more about social risk factors and to identify areas for targeted interventions. For clinical translation, it can be beneficial for OB/GYN providers to have insight into specific subgroups of women who are at greatest risk of low nutrition literacy, for whom tailored counseling and public health programs can be created. Therefore, the objectives of our study are to determine the level of nutrition literacy among Chinese women aged 18–49 years using the CNLQ and examine the socio-demographic and behavioral correlates.

## 2 Materials and methods

### 2.1 Study design and participants

A cross-sectional, online survey was conducted from August 20, 2024 to March 20, 2025. Participants were recruited by the research team with dissemination supported by three tertiary hospitals in different regions of China, including one hospital in East China, one in North China, and one in Middle China. Survey dissemination was facilitated through the professional and personal networks of healthcare professionals affiliated with the participating hospitals. Eligibility was restricted to women aged 18–49 years. The minimum age of 18 years was set to ensure legal capacity to provide informed consent, and the upper age of 49 years was selected to align with the reproductive age range commonly used in population-based health research in China.

Participants were eligible if they (1) were female; (2) were 18–49 years of age; (3) had Chinese nationality and resided in mainland China; (4) could read and understand Chinese; (5) provided electronic informed consent; and (6) completed the entire questionnaire. Exclusion criteria were (1) male respondents; (2) age outside 18–49 years; (3) patterned or clearly invalid responses (for example, selecting the same option for all questions or implausible demographic entries); (4) duplicate submissions from the same IP address; and (5) current hospitalization or serious illness that could compromise response validity.

The questionnaire was hosted on an online survey platform and accessed via a QR code and survey link. The QR code and link were distributed through WeChat Moments, QQ Space, and WeChat and QQ messages and groups. Participation was voluntary. An electronic informed consent statement was presented at the beginning of the survey, and participants proceeded only after indicating consent. All items were required before submission, hence incomplete questionnaires being not possible.

The minimum sample size was calculated using typical multiple regression standard formulas for a medium effect size and 17 predictor variables, with power of 0.80 and alpha of 0.05. The estimate was 325 participants as a minimum. With the number of potential exclusions in mind and to allow for good statistical precision for subgroup analyses, the final sample recruitment of 1,391 far exceeded expectations and allowed good power for multivariable modeling.

### 2.2 Instrument

Nutrition literacy was assessed using the Chinese Nutrition Literacy Questionnaire (CNLQ) which has been validated for use in a general Chinese population. The CNLQ is structured based on functional, interactive and critical nutrition literacy. It has shown favorable psychometric properties in previous studies, with Cronbach’s *α* coefficients of 0.93 for the total scale and between 0.82 and 0.89 within its subscales. The 3-factor model showed good fit indices (CFI > 0.90, RMSEA < 0.08) in the confirmatory factor analysis (23).

For the present study, the CNLQ wording was minimally adapted to improve relevance for women of childbearing age by replacing generic phrasing with women-of-childbearing-age relevant phrasing, while maintaining the original structure, item content, response options, and scoring protocol. Domain and total scores were calculated according to the original CNLQ scoring protocol and transformed to a 0 to 100 scale. Higher scores indicate higher nutrition literacy. Adequate nutrition literacy was defined as a total score of 80 points or higher, which was adopted from the original validation study as an *a priori* benchmark for high proficiency (23). See Supplementary Table S1 for the CNLQ used.

### 2.3 Outcomes and covariates

Primary outcomes were (1) total nutrition literacy score as a continuous variable and (2) adequate nutrition literacy as a binary variable defined by total score ≥80. Functional, interactive, and critical domain scores were summarized descriptively and compared across participant subgroups.

Socio-demographic and health-related variables collected included age, ethnic group, area of residence, education level, marital status, healthcare background, average monthly household income per person, chronic disease status, and body mass index (BMI). Income categories were determined empirically by the research team based on the observed distribution and to ensure adequate numbers in each group for stable estimation.

### 2.4 Statistical analysis

All analyses were performed using SPSS version 20.0 (IBM Corp., Armonk, NY, USA). Continuous variables were summarized as mean ± standard deviation (SD) when approximately normally distributed; otherwise, median with interquartile range (IQR) was reported. Categorical variables were summarized as frequency and percentage. The Shapiro–Wilk test and visual inspection of histograms were used to evaluate distributional assumptions for continuous variables. Group comparisons for the total nutrition literacy score used independent-samples t tests or one-way ANOVA when assumptions were met, and nonparametric alternatives were used when appropriate. For categorical comparisons, chi-square tests were applied.

Univariable screening was used for predictor selection prior to multivariable modeling. For the continuous outcome (total nutrition literacy score), each candidate predictor was first evaluated in a univariable linear regression model. For the binary outcome (adequate nutrition literacy), each candidate predictor was first evaluated in a univariable logistic regression model. Predictors with *p* < 0.10 in the corresponding univariable analysis were entered into the multivariable model for that outcome. No predictors were forced into multivariable models.

Multivariable linear regression was then conducted to estimate adjusted associations with total nutrition literacy score, and multivariable logistic regression was conducted to estimate adjusted odds ratios (AORs) with 95% confidence intervals for adequate nutrition literacy. Age was categorized into 18 to 24, 25 to 34, and 35 to 49 years for regression analyses. Multicollinearity among predictors retained in each multivariable model was assessed using variance inflation factors (VIF), with VIF < 5 considered acceptable. For linear regression, linearity and homoscedasticity were evaluated using residual plots, and residual normality was assessed using graphical methods. For logistic regression, model fit was evaluated using the Hosmer–Lemeshow goodness-of-fit test, and model discrimination was summarized using standard classification performance indices as appropriate.

All tests were two-sided, and *p* < 0.05 was considered statistically significant. Numerical reporting was standardized across tables: regression coefficients and confidence intervals were presented to two decimal places, and *p*-values were presented to three decimal places, reporting *p* < 0.001 when applicable.

### 2.5 Ethical considerations and informed consent

The study was conducted in accordance with the Declaration of Helsinki, and was ethically approved by the Ethics Committee of Dushu Lake Hospital of Suzhou (Approval number 241076). Electronic informed consent was obtained at the beginning of the questionnaire; submission indicated consent to participate.

## 3 Results

### 3.1 Participant recruitment and analytic sample

**Figure 1:**
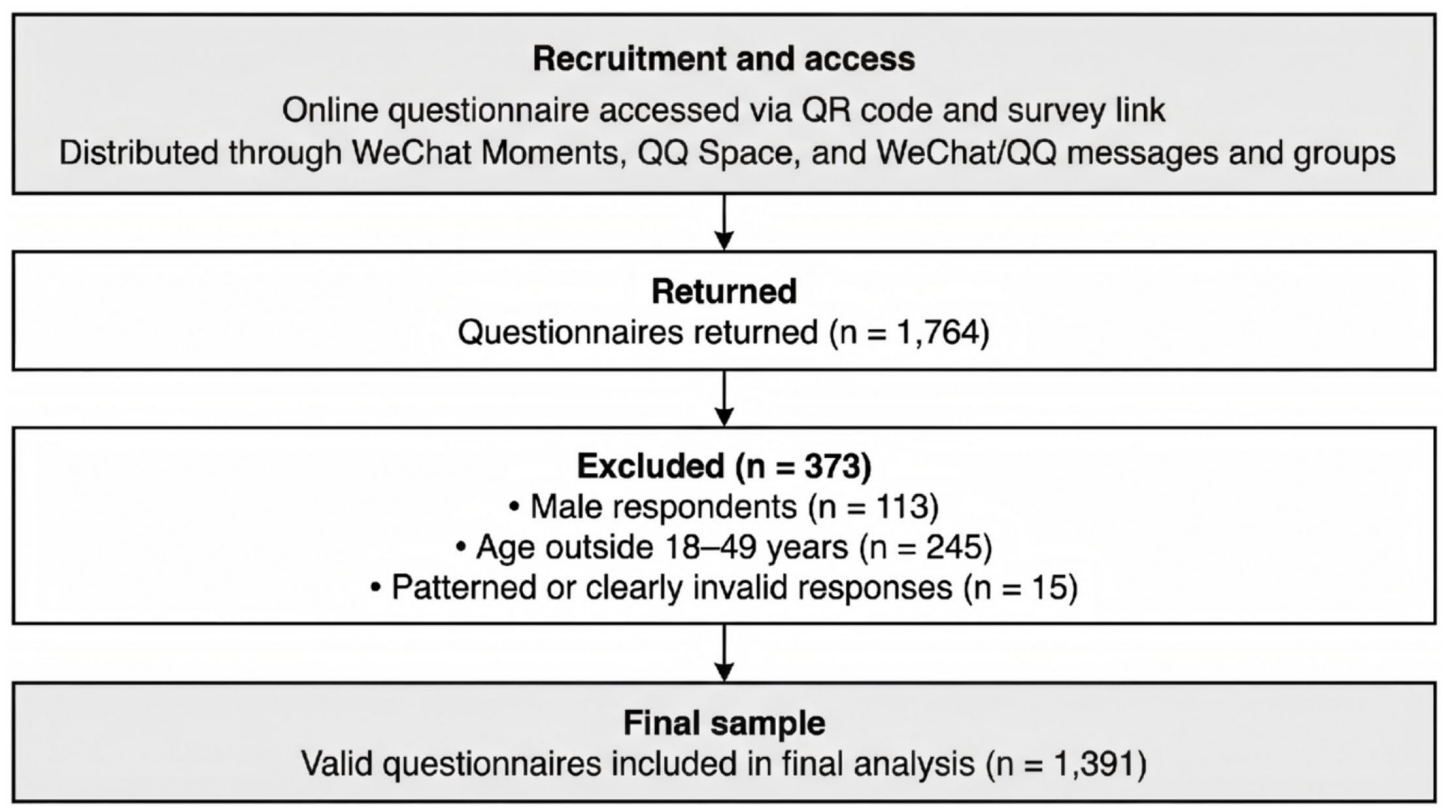
Flow of participant recruitment and data screening. The online questionnaire was accessed via a QR code and survey link disseminated through WeChat Moments, QQ Space, and WeChat or QQ messages and groups. Participation was voluntary, and completion of all items was required for submission. Of the 1,764 returned questionnaires, 373 were excluded for male respondents (*n* = 113), age outside 18–49 years (*n* = 245), and patterned or clearly invalid responses (*n* = 15), resulting in 1,391 valid questionnaires included in the final analysis.

A total of 1,764 questionnaires were returned. After data screening, 373 questionnaires were excluded for male respondents (*n* = 113), age outside 18–49 years (*n* = 245), and patterned or clearly invalid responses (*n* = 15), resulting in a final analytic sample of 1,391 valid questionnaires (Figure 1).

### 3.2 Participant characteristics and nutrition literacy score

Table 1 summarizes participant characteristics. The mean age was 33.8 ± 7.8 years. Participants were distributed across age categories as follows: 19.6% were 18–24 years (*n* = 273), 41.8% were 25–34 years (*n* = 582), and 38.6% were 35–49 years (*n* = 536). Most participants were Han (93.4%, *n* = 1,299) and lived in urban areas (68.6%, *n* = 954). Regarding education, over half had a college education or above (57.4%, *n* = 799), 29.9% had senior high school education (*n* = 416), and 12.7% had junior high education or below (*n* = 176). Most participants were married (69.0%, *n* = 960), while 24.5% were never married (*n* = 341) and 6.5% were divorced or other (*n* = 90). Healthcare background was reported by 27.2% of participants (*n* = 378).

**Table 1:** Socio-demographic and health characteristics of participants (*N* = 1,391).

Characteristic	*n* (%) or Mean ± SD
Age (years)	33.8 ± 7.8
18–24	273 (19.6)
25–34	582 (41.8)
35–49	536 (38.6)
Ethnicity	
Han	1,299 (93.4)
Other	92 (6.6)
Residence	
Urban	954 (68.6)
Rural	437 (31.4)
Education	
≤ Junior high	176 (12.7)
Senior high	416 (29.9)
College or above	799 (57.4)
Marital status	
Never married	341 (24.5)
Married	960 (69.0)
Divorced/other	90 (6.5)
Healthcare background	
Yes	378 (27.2)
No	1,013 (72.8)
Income (CNY)	
≤ 5,000	196 (14.1)
5,001–8,000	357 (25.7)
8,001–13,000	401 (28.8)
13,001–17,000	215 (15.5)
17,001–24,000	129 (9.3)
> 24,000	93 (6.7)
Chronic disease	
None	1,014 (72.9)
Single	272 (19.6)
Multi	105 (7.5)
BMI category	
Underweight (<18.5)	128 (9.2)
Normal (18.5–23.9)	865 (62.2)
Overweight (24.0–27.9)	286 (20.6)
Obese (≥28.0)	112 (8.1)

Monthly household income per person was most commonly 8,001–13,000 CNY (28.8%, *n* = 401) and 5,001–8,000 CNY (25.7%, *n* = 357), followed by 13,001–17,000 CNY (15.5%, *n* = 215) and ≤5,000 CNY (14.1%, *n* = 196). Higher income categories were less frequent, with 9.3% reporting 17,001–24,000 CNY (*n* = 129) and 6.7% reporting >24,000 CNY (*n* = 93). Most participants reported no chronic disease (72.9%, *n* = 1,014); 19.6% reported a single chronic disease (*n* = 272), and 7.5% reported multimorbidity (*n* = 105). For BMI, 62.2% were in the normal range (18.5–23.9 kg/m^2^; *n* = 865), 20.6% were overweight (24.0–27.9 kg/m^2^; *n* = 286), 9.2% were underweight (<18.5 kg/m^2^; *n* = 128), and 8.1% were obese (≥28.0 kg/m^2^; *n* = 112).

### 3.3 Total and domain-specific nutrition literacy

Table 2 presents the overall and domain-specific nutrition literacy scores. The mean total nutrition literacy score was 74.9 ± 12.8 on the 0–100 scale. Using the predefined adequacy threshold (total score ≥80), 33.4% of participants (*n* = 465) were classified as having adequate nutrition literacy. Domain-specific results showed a consistent profile: functional nutrition literacy was highest, interactive nutrition literacy was intermediate, and critical nutrition literacy was lowest.

**Table 2:** Total and domain-specific nutrition literacy scores (*N* = 1,391).

Outcome (0–100)	Mean ± SD	Median (Q1–Q3)
Functional nutrition literacy	77.4 ± 13.4	79 (69–88)
Interactive nutrition literacy	75.1 ± 13.8	76 (66–85)
Critical nutrition literacy	69.9 ± 14.2	71 (60–81)
Total nutrition literacy	74.9 ± 12.8	76 (66–85)

Adequate nutrition literacy (total score ≥80): 465/1,391 (33.4%).

### 3.4 Univariable regression analyses

Univariable linear regression was used to screen candidate predictors for the continuous outcome (total nutrition literacy score). Predictors meeting the prespecified entry criterion (*p* < 0.10) were eligible for inclusion in the multivariable linear regression model. In univariable analyses, age group, residence, education, marital status (married vs. never married), healthcare background, multiple income categories, multimorbidity, and BMI categories (overweight and obese) met the entry criterion. Ethnicity, single chronic disease, and underweight BMI did not meet the entry criterion and were not advanced to multivariable modeling. (Table 3).

**Table 3:** Univariable linear regression screening for total nutrition literacy score (*N* = 1,391).

Predictor	β (95% CI)	*p*-value
Age group (ref: 17–23)		
25–34	1.94 (0.53 to 3.35)	0.007
35–49	3.21 (1.78 to 4.63)	<0.001
Ethnicity (ref: Other)		
Han	0.84 (−1.47 to 3.15)	0.476
Residence (ref: Rural)		
Urban	4.06 (2.98 to 5.15)	<0.001
Education (ref: ≤Junior high)		
Senior high	3.87 (2.41 to 5.33)	<0.001
College or above	8.52 (7.10 to 9.93)	<0.001
Marital status (ref: Never married)		
Married	1.12 (−0.21 to 2.45)	0.099
Divorced/other	−0.48 (−2.38 to 1.43)	0.624
Healthcare background (ref: No)		
Yes	3.06 (1.95 to 4.17)	<0.001
Income per person (CNY) (ref: ≤5,000)		
5,001–8,000	1.08 (−0.03 to 2.19)	0.057
8,001–13,000	2.63 (1.42 to 3.84)	<0.001
13,001–17,000	4.09 (2.62 to 5.56)	<0.001
17,001–24,000	4.78 (3.00 to 6.56)	<0.001
>24,000	6.31 (4.13 to 8.49)	<0.001
Chronic disease status (ref: None)		
Single	−0.94 (−2.30 to 0.42)	0.176
Multi	−3.48 (−5.28 to −1.68)	<0.001
BMI category (ref: Normal)		
Underweight	−0.32 (−2.05 to 1.41)	0.717
Overweight	−1.41 (−2.69 to −0.14)	0.030
Obese	−2.16 (−3.93 to −0.39)	0.017

*β* is the unstandardized regression coefficient for difference in total score relative to the reference group.

Univariable logistic regression was used to screen predictors for the binary outcome of adequate nutrition literacy (total score ≥80). Predictors meeting the prespecified entry criterion (*p* < 0.10) were eligible for inclusion in the multivariable logistic regression model. Age group, residence, education, marital status (married vs. never married), healthcare background, higher income categories (≥8,001 CNY), multimorbidity, and obesity met the entry criterion. Ethnicity, single chronic disease, and underweight/overweight BMI did not meet the entry criterion. (Table 4).

**Table 4:** Univariable logistic regression screening for adequate nutrition literacy (total score ≥80) (*N* = 1,391).

Predictor	OR (95% CI)	*p*-value
Age group (ref: 17–23)		
25–34	1.29 (1.01 to 1.66)	0.041
35–49	1.74 (1.34 to 2.26)	<0.001
Ethnicity (ref: Other)		
Han	1.08 (0.69 to 1.68)	0.735
Residence (ref: Rural)		
Urban	2.02 (1.62 to 2.52)	<0.001
Education (ref: ≤Junior high)		
Senior high	1.93 (1.39 to 2.69)	<0.001
College or above	3.68 (2.67 to 5.07)	<0.001
Marital status (ref: Never married)		
Married	1.24 (0.99 to 1.55)	0.061
Divorced/other	0.92 (0.56 to 1.51)	0.742
Healthcare background (ref: No)		
Yes	1.77 (1.40 to 2.25)	<0.001
Income per person (CNY) (ref: ≤5,000)		
5,001–8,000	1.17 (0.88 to 1.54)	0.275
8,001–13,000	1.49 (1.12 to 1.98)	0.006
13,001–17,000	1.86 (1.27 to 2.72)	0.001
17,001–24,000	2.12 (1.36 to 3.31)	0.001
>24,000	2.58 (1.51 to 4.41)	<0.001
Chronic disease status (ref: None)		
Single	0.89 (0.69 to 1.16)	0.391
Multi	0.55 (0.35 to 0.86)	0.008
BMI category (ref: Normal)		
Underweight	0.99 (0.66 to 1.49)	0.965
Overweight	0.88 (0.70 to 1.10)	0.258
Obese	0.72 (0.50 to 1.03)	0.073

OR > 1 indicates higher odds of adequate literacy relative to the reference group.

### 3.5 Multivariable linear regression analyses

Predictors meeting the univariable entry criterion (*p* < 0.10) were entered into the multivariable linear regression model for total nutrition literacy score. After adjustment, urban residence, higher education, higher income, and having a healthcare background remained independently associated with higher total nutrition literacy. Multimorbidity remained negatively associated with total score. BMI obesity was independently associated with a lower score, whereas overweight showed a borderline association. The association for marital status did not persist after adjustment. No evidence of problematic multicollinearity was observed (all VIFs <2.50). (Table 5).

**Table 5:** Multivariable linear regression for total nutrition literacy score (*N* = 1,391).

Predictor	Adjusted *β* (95% CI)	*p*-value
Age group (ref: 17–23)		
25–34	0.83 (−0.44 to 2.10)	0.201
35–49	1.67 (0.29 to 3.05)	0.018
Residence (ref: Rural)		
Urban	2.47 (1.41 to 3.53)	<0.001
Education (ref: ≤Junior high)		
Senior high	3.02 (1.60 to 4.44)	<0.001
College or above	6.82 (5.31 to 8.33)	<0.001
Marital status (ref: Never married)		
Married	0.36 (−0.78 to 1.50)	0.536
Healthcare background (ref: No)		
Yes	2.05 (0.96 to 3.14)	<0.001
Income per person (CNY) (ref: ≤5,000)		
5,001–8,000	0.44 (−0.67 to 1.55)	0.438
8,001–13,000	1.51 (0.32 to 2.70)	0.013
13,001–17,000	2.58 (1.11 to 4.05)	0.001
17,001–24,000	3.04 (1.30 to 4.78)	0.001
>24,000	4.33 (2.18 to 6.48)	<0.001
Chronic disease status (ref: None)		
Multi	−2.55 (−4.29 to −0.81)	0.004
BMI category (ref: Normal)		
Overweight	−1.03 (−2.26 to 0.20)	0.100
Obese	−1.84 (−3.56 to −0.12)	0.036

*F*-test, *p* < 0.001; *R*^2^ = 0.30; adjusted *R*^2^ = 0.29.

Predictors meeting the univariable entry criterion (*p* < 0.10) were entered into the multivariable logistic regression model for adequate nutrition literacy (total score ≥80). After adjustment, urban residence, higher education, higher income, and healthcare background remained independently associated with higher odds of adequate nutrition literacy. Multimorbidity remained associated with lower odds of adequacy. The association for obesity attenuated and was not statistically significant after adjustment. Model calibration was acceptable (Hosmer–Lemeshow *p* = 0.472), and multicollinearity was not evident (all VIFs < 2.30). (Table 6).

**Table 6:** Multivariable logistic regression for adequate nutrition literacy (total score ≥80) (*N* = 1,391).

Predictor	Adjusted OR (95% CI)	*p*-value
Age group (ref: 17–23)		
25–34	1.16 (0.90 to 1.50)	0.253
35–49	1.43 (1.06 to 1.93)	0.019
Residence (ref: Rural)		
Urban	1.64 (1.29 to 2.09)	<0.001
Education (ref: ≤Junior high)		
Senior high	1.66 (1.15 to 2.40)	0.007
College or above	2.93 (2.08 to 4.12)	<0.001
Marital status (ref: Never married)		
Married	1.09 (0.86 to 1.38)	0.485
Healthcare background (ref: No)		
Yes	1.42 (1.11 to 1.82)	0.005
Income per person (CNY) (ref: ≤5,000)		
8,001–13,000	1.28 (0.97 to 1.70)	0.082
13,001–17,000	1.52 (1.03 to 2.25)	0.035
17,001–24,000	1.76 (1.11 to 2.80)	0.017
>24,000	2.05 (1.18 to 3.55)	0.011
Chronic disease status (ref: None)		
Multi	0.62 (0.39 to 0.98)	0.042
BMI category (ref: Normal)		
Obese	0.79 (0.54 to 1.15)	0.214

Likelihood ratio *χ*^2^, *p* < 0.001; Nagelkerke *R*^2^ = 0.25; Hosmer–Lemeshow *p* = 0.472.

## 4 Discussion

### 4.1 Principal findings

In this cross-sectional online survey, we assessed nutrition literacy among Chinese women of childbearing age. Three principal findings are notable. First, overall nutrition literacy was moderate, with one-third of participants meeting the adequacy threshold (total score ≥80). Second, domain scores showed a clear gradient, with functional nutrition literacy highest, interactive nutrition literacy intermediate, and critical nutrition literacy lowest. Third, higher education, higher income, urban residence, and having a healthcare background were associated with higher nutrition literacy, while multimorbidity was consistently associated with lower nutrition literacy and lower odds of adequacy.

### 4.2 Interpretation in the context of existing literature

The overall nutrition literacy level observed is consistent with patterns reported in prior research where nutrition and health literacy are socially patterned rather than evenly distributed across populations (24–26). Although direct numerical comparisons across studies should be interpreted cautiously because of differences in instruments and sampling approaches, the directions of association observed in this study align with a well-established social gradient (27, 28). Education and income likely reflect both access to health information and the skills required to understand and apply nutrition knowledge (29, 30). Urban residence may capture greater exposure to structured health promotion activities, more frequent contact with healthcare services, and improved access to credible nutrition information channels compared with rural settings (31, 32).

The positive association with healthcare background is also expected (33). Individuals with healthcare exposure are more likely to encounter nutrition information through training, workplace norms, and professional networks, and may have greater familiarity with evidence-based recommendations (34, 35). This pattern supports the construct validity of the instrument in this population because groups expected to have higher nutrition literacy demonstrated higher scores and higher adequacy prevalence (36).

### 4.3 Why critical nutrition literacy was lowest

A key contribution of this study is the explicit domain-level characterization of nutrition literacy. Critical nutrition literacy was the lowest scoring domain, which is consistent with broader health literacy literature (37–39). Functional literacy relates to foundational knowledge and comprehension of routine nutrition guidance, while interactive literacy reflects information-seeking and communication skills that can be facilitated by widespread smartphone access and social media use (40, 41). Critical literacy requires higher-order skills: evaluating the credibility of sources, distinguishing evidence-based guidance from marketing claims, reconciling conflicting advice, and making decisions under uncertainty (42–44). These competencies are more demanding and are less likely to develop through passive exposure to general nutrition messages (39, 45).

The current information environment may intensify this gap. Women of childbearing age are frequently exposed to large volumes of nutrition content through short-video platforms, influencers, and commercial advertising, where persuasive narratives can be presented with minimal evidentiary support (46, 47). In such contexts, individuals may be able to recognize general “healthy eating” messages but still struggle to judge reliability, identify conflicts of interest, or interpret contradictory claims (48, 49). This pattern suggests that interventions should not focus solely on increasing nutrition knowledge, but should explicitly target critical appraisal skills and decision-making (44, 50).

### 4.4 Multimorbidity and BMI as indicators of vulnerability

Multimorbidity was consistently associated with lower total nutrition literacy scores and lower odds of meeting the adequacy threshold (51, 52). Several mechanisms may explain this relationship. First, multimorbidity increases the complexity of dietary recommendations. Individuals with multiple chronic conditions may receive overlapping or even conflicting advice, making it more difficult to integrate guidance into coherent daily decisions, particularly when critical literacy is limited (53, 54). Second, multimorbidity can be accompanied by time constraints, treatment burden, stress, and reduced cognitive bandwidth for preventive behaviors, which may hinder sustained engagement with nutrition information (55). Third, increased healthcare contact does not necessarily translate into improved literacy if clinical encounters prioritize acute management and medication over structured patient education or if counseling messages are inconsistent (56).

Higher BMI, particularly obesity, was associated with lower total nutrition literacy scores in adjusted analyses (39, 57). This association is plausible because lower nutrition literacy may contribute to less informed food choices and reduced ability to interpret nutrition information, while obesity may also be linked to exposure to misinformation and cyclical engagement with fad diets (2, 58). Importantly, obesity was not independently associated with adequate nutrition literacy after adjustment, suggesting that the relationship may be more apparent when literacy is treated as a continuous skill gradient than when dichotomized at a single adequacy threshold (59, 60).

### 4.5 Age and life-course exposure

Age-group associations were present in both outcomes, but the pattern differed by group. After adjustment, women aged 35–49 years had higher total nutrition literacy scores and higher odds of adequate literacy compared with those aged 18–24 years, whereas the association for ages 25–34 years was attenuated (61, 62). This may reflect cumulative exposure to nutrition information through life-course experiences such as pregnancy-related counseling, child-rearing, family caregiving, and repeated contact with healthcare services (63, 64). However, because age correlates with socioeconomic position and health status, these findings likely reflect combined contextual and experiential influences rather than age effects alone.

### 4.6 Implications for policy and practice

Our findings support targeted and domain-specific strategies to improve nutrition literacy among women of childbearing age. First, interventions should explicitly incorporate critical nutrition literacy components, including how to evaluate information sources, identify misleading marketing, interpret health claims, and apply simple credibility heuristics aligned with authoritative guidance. Second, population subgroups associated with lower literacy in this study, particularly rural residents, women with lower education or income, and women with multimorbidity, may benefit from tailored delivery formats that reduce cognitive load and emphasize actionable skills rather than dense information. For rural communities, integration into primary care and community health services may be appropriate. For lower education groups, plain-language materials, visual aids, and decision supports may improve comprehension and application.

Third, the association with healthcare background suggests that healthcare settings can be leveraged to disseminate evidence-based nutrition information, but standardized and consistent messaging is essential to avoid confusion. Incorporating brief, structured nutrition counseling into routine women’s health services and chronic disease management may be particularly relevant for women with multimorbidity. Finally, given the central role of digital media in nutrition information exposure, partnerships between public health agencies, professional societies, and digital platforms may help disseminate credible information and improve appraisal skills, rather than relying only on prescriptive dietary tips.

### 4.7 Strengths and limitations

This study has several strengths. It included a large sample size exceeding the *a priori* minimum requirement, recruitment supported by three tertiary hospitals in different regions of China, and assessment using a validated instrument with minimal wording changes and psychometric evaluation in the full analytic sample. Reporting domain-specific scores offers a more actionable profile than reporting a total score alone.

Limitations should be considered. First, the cross-sectional design precludes causal inference and bidirectional relationships are likely. Second, recruitment relied on online dissemination through hospital-affiliated professional networks and social media channels, which may over-represent women with better digital access or stronger health engagement, potentially limiting generalizability. Third, measures were self-reported, which may introduce misclassification for chronic disease status and BMI. Fourth, although the adapted instrument showed acceptable psychometric performance in this sample, measurement invariance across key subgroups (for example, education or urban versus rural residence) was not assessed and warrants future study. Fifth, the covariate selection approach used univariable screening prior to multivariable modeling; while this approach improves parsimony, it can omit important confounders, and future work could evaluate theory-driven adjustment as a sensitivity analysis.

## 5 Conclusion

Among Chinese women of childbearing age, overall nutrition literacy is moderate, with about one-third meeting the adequacy threshold. Critical nutrition literacy is the lowest scoring domain, indicating a distinct gap in appraisal and evaluation skills in an information-dense environment. Higher education, higher income, urban residence, and healthcare background are associated with higher nutrition literacy, whereas multimorbidity is associated with lower literacy and lower odds of adequate literacy. Interventions should prioritize critical literacy skills and focus on structurally disadvantaged groups and women with multimorbidity, using tailored formats and consistent evidence-based messaging across healthcare and digital platforms.

## Data Availability

The raw data supporting the conclusions of this article will be made available by the authors, without undue reservation.
