# Supplementary material for: Nutrition literacy and socio-demographic determinants among Chinese women of childbearing age
Source: Front Public Health. 2026 Feb 24;14:1742415. doi: 10.3389/fpubh.2026.1742415 (PMC12974174; doi:10.3389/fpubh.2026.1742415)
Supplement: Supplementary file 1 [file Table_1.docx]

**中国育龄期妇女营养素养及其影响因素调查问卷**

## **致受访者的一封信**

您好！

我们正在开展一项题为“育龄期妇女营养素养及其影响因素的调查研究”的在线问卷调查。本研究旨在了解中国育龄期妇女（18～49岁）的营养素养水平及其社会人口学与行为学影响因素，为今后制定营养健康促进措施提供科学依据。

本问卷由研究团队独立开展，数据仅用于科研分析与学术发表。问卷填写完全匿名，不会收集可识别个人身份的信息。您的参与完全自愿，您可以在任何时候退出问卷而不产生任何不良后果。所有数据将严格保密，仅供本研究使用。

本问卷预计耗时约15–20分钟。若您符合调查条件并选择继续填写与提交问卷，即表示您已充分了解本研究的目的、内容及保密原则，并自愿同意参加本次调查。

感谢您的支持与配合！祝您身体健康！

1.您出生于[下拉菜单]*

______年_______月________日（公历）

*注：逻辑设置：若计算年龄 < 18 或 > 49 → 显示提示“感谢您的参与，本调查仅针对18–49岁的女性。” → 结束问卷。*

2.性别[单选题]*

| ①男（注：结束问卷，展示消息“感谢您的参与，本调查仅针对女性受访者。”） |
| --- |
| ②女（注：继续答题） |

3.民族[单选题]*

| ①汉族 |
| --- |
| ②其他民族_________________* |

4.（1）您的常住地属于[单选题]*

| ①城镇 |
| --- |
| ②农村 |

4.（2）地址[填空题]*

_______省________市________县（区）

5.您的教育程度[单选题]*

| ①初中或以下 | ②高中 |
| --- | --- |
| ③大学本科/大专或以上 |  |

6.您的婚姻状况[单选题]*

| ①未婚 |
| --- |
| ②已婚 |
| ③离异 |
| ④其他 |

7.您的职业[单选题]*

| ①农民 | ②工人 |
| --- | --- |
| ③机关/事业单位职员 | ④企业员工 |
| ⑤商业服务业人员 | ⑥个体户 |
| ⑦军人、武警人员 | ⑧演员、运动员 |
| ⑨学生 | ⑩其他 |

8.您是否接受过医疗卫生相关的教育或有过相关工作经历[单选题]*

| ①是 |
| --- |
| ②否 |

9.您家庭的人均月收入（人均月收入=家庭总收入/家庭成员总人数，家庭成员包括小孩和老人）状况[单选题]*

| ①不超过5千/月 |
| --- |
| ②超过5千/月，不超过8千/月 |
| ③超过8千/月，不超过1.3万/月 |
| ④超过1.3万/月，不超过1.7万/月 |
| ⑤超过1.7万/月，不超过2.4万/月 |
| ⑥超过2.4万/月 |

10.您是否患有以下慢性疾病[矩阵多选题]*

|  | 是 | 否 |
| --- | --- | --- |
| 血脂异常（高血脂或低血脂） | □ | □ |
| 血糖升高或糖尿病 | □ | □ |
| 高血压 | □ | □ |
| 癌症或恶性肿瘤 | □ | □ |
| 慢性肺部疾病（如支气管炎、肺气肿） | □ | □ |
| 肝脏疾病 | □ | □ |
| 心脏病 | □ | □ |
| 肾脏疾病（不包括肿瘤或癌症） | □ | □ |
| 关节炎或风湿病 | □ | □ |

 11.您的[矩阵文本题]*

|  |  |
| --- | --- |
| 身高是（cm） | ________________________ |
| 体重是（kg） | ________________________ |

12.您是否赞同以下说法？[矩阵多选题]*

|  |  | 非常赞同 | 赞同 | 一般 | 不赞同 | 非常不赞同 |
| --- | --- | --- | --- | --- | --- | --- |
| 1 | 合理膳食是维系健康、远离疾病的重要基础。 | □ | □ | □ | □ | □ |
| 2 | 良好的膳食模式是保障营养充足的基础。 | □ | □ | □ | □ | □ |
| 3 | 食物分为五大类，包括谷薯类、蔬菜水果类、畜禽鱼蛋奶类、大豆坚果类和油脂类，不同食物具有不同的营养学特点。 | □ | □ | □ | □ | □ |
| 4 | 全谷物属于谷薯类。 | □ | □ | □ | □ | □ |
| 5 | 日常生活中，我们要努力做到食不过量，吃动平衡。 | □ | □ | □ | □ | □ |
| 6 | 各年龄阶段的人群都应该天天运动，保持健康体重。 | □ | □ | □ | □ | □ |
| 7 | 我们应该珍惜食物，按需备餐，杜绝浪费。 | □ | □ | □ | □ | □ |
| 8 | “分餐”、“份餐”是平衡膳食、简约就餐的好形式。 | □ | □ | □ | □ | □ |
| 9 | 我们应该尊重他人饮食习俗，注重餐桌礼仪。 | □ | □ | □ | □ | □ |
| 10 | 营养健康餐饮文明是饮食文明的核心。 | □ | □ | □ | □ | □ |
| 11 | 和家人朋友一起就餐可促进幸福感，也能促进食欲。 | □ | □ | □ | □ | □ |
| 12 | 家庭是良好饮食文化传统传承的最佳场所。 | □ | □ | □ | □ | □ |

13.请您回忆一下最近一周所吃的食物，根据实际情况勾选平均每天吃以下食物的种类。（例如：小明昨天吃了馒头、米饭、芸豆、红薯，则他吃了4种谷薯杂豆类食物；他还吃了苹果、大白菜、西红柿，则他吃了3种蔬菜水果类食物）

以下是食物类别及种类数量：

[矩阵单选题]*

|  | 是 | 否 |
| --- | --- | --- |
| 谷薯杂豆≥3 |  |  |
| 蔬菜水果≥4 |  |  |
| 畜禽鱼蛋≥3 |  |  |
| 奶豆坚果≥2 |  |  |

14.请您回忆一下最近一周生活内容，按照您的实际情况回答下列问题：[矩阵单选题]*

| 您过去一周内有几天吃了以下食物？ | 每天 | 5～6天 | 3～4天 | 1～2天 | 0天 |
| --- | --- | --- | --- | --- | --- |
| 1. 早餐 |  |  |  |  |  |
| 1. 粗杂粮（薯类、杂豆、玉米、燕麦、糙米等） |  |  |  |  |  |
| 1. 蔬菜超过300g（6两） |  |  |  |  |  |
| 1. 水果 |  |  |  |  |  |
| 1. 牛奶、酸奶、奶粉或奶酪（不包括优酸乳、营养快线等乳饮料） |  |  |  |  |  |
| 1. 鱼/禽/蛋/瘦肉 |  |  |  |  |  |
| 1. 含糖食品（如面包、蛋糕、饼干等零食） |  |  |  |  |  |
| 1. 油炸、烧烤、膨化食品 |  |  |  |  |  |

15.您过去一周平均每天喝多少杯水？（1杯=200ml）[单选题]*

| ①7-8杯 |
| --- |
| ②5-6杯 |
| ③3-4杯 |
| ④1-2杯 |
| ⑤不知道 |

16.您平时喝酒的情况？[单选题]*

| ①总是喝酒 |
| --- |
| ②经常喝酒 |
| ③有时喝酒 |
| ④极少喝酒 |
| ⑤完全不喝酒 |

17.过去一个月，您每周有几天的累计户外运动时间超过30分钟？（不包括上下班、买菜、走路的运动时间）[单选题]*

| ①每天 |
| --- |
| ②5-6天 |
| ③3-4天 |
| ④1-2天 |
| ⑤0天 |

18.您对“估算食物份量和合理搭配食物是必备的营养技能”的态度是？[单选题]*

| ①非常赞同 |
| --- |
| ②赞同 |
| ③一般 |
| ④不赞同 |
| ⑤非常不赞同 |

19.一个乒乓球大小的鸡蛋的重量大约为多少？[单选题]*

| ①5g |
| --- |
| ②50g（一两） |
| ③100g（二两） |
| ④500g（一斤） |
| ⑤不知道 |

20.下面是几位成人的早餐清单，您认为哪一位的早餐搭配最合理？[单选题]*

| ①豆浆、全麦面包、咸菜 |
| --- |
| ②豆浆、油条、咸菜 |
| ③牛奶、油条、水果 |
| ④牛奶、全麦面包、水果 |
| ⑤不知道 |

21.食品营养标签指的是在食品的外包装上标注营养成分并显示营养信息，以及适当的营养声称和健康声明。请问您在购买包装食品时，会看食品营养标签吗？[单选题]

| ①完全不会 |
| --- |
| ②极少会 |
| ③有时会 |
| ④经常会 |
| ⑤总是会 |

22.您平时在购买食品时，会优先购买不含反式脂肪酸的食品吗？[单选题]*

| ①完全不会 |
| --- |
| ②极少会 |
| ③有时会 |
| ④经常会 |
| ⑤总是会 |

23.在食品营养标签中，“营养素参考值%/NRV%”是指100克/毫升该食品所含营养成分占每日参考摄入量的百分比。那么，如果只靠摄入该食品来摄入蛋白质，那喝多少就可以满足我们一天需要的蛋白质？（估算即可）


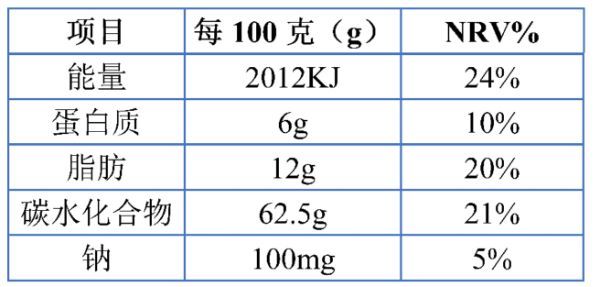


[单选题]*

| ①400g |
| --- |
| ②600g |
| ③800g |
| ④1000g |
| ⑤不知道 |

24.您在超市购买合格生肉时最看重的是？[单选题]*

| ①新鲜度 |
| --- |
| ②价格 |
| ③包装 |
| ④品牌 |
| ⑤产地 |

25.您平时会使用，或家中做饭的人会使用烧烤煎炸的烹调方式吗？[单选题]*

| ①完全不会 |
| --- |
| ②极少会 |
| ③有时会 |
| ④经常会 |
| ⑤总是会 |

26.您平时在点外卖和在外就餐的时候会考虑烹调方式吗？[单选题]*

| ①完全不会 |
| --- |
| ②极少会 |
| ③有时会 |
| ④经常会 |
| ⑤总是会 |

27.您平时会关注营养信息吗？[单选题]*

| ①完全不会 |
| --- |
| ②极少会 |
| ③有时会 |
| ④经常会 |
| ⑤总是会 |

28.面对各种渠道来源的营养知识与信息，您会怎么做？[单选题]*

| ①全盘接受 |
| --- |
| ②进行甄别后选择性接受 |
| ③完全不接受 |
| ④根本无法获得营养知识与信息 |
| ⑤不知道怎么办 |

29.您接收到有用的营养知识与信息后，会分享给身边的人吗？[单选题]*

| ①完全不会 |
| --- |
| ②极少会 |
| ③有时会 |
| ④经常会 |
| ⑤总是会 |

30.您对“保健（功能）食品可以代替药品”的态度是？ [单选题]*

| ①非常赞同 |
| --- |
| ②赞同 |
| ③一般 |
| ④不赞同 |
| ⑤非常不赞同 |

31.您对“保健（功能）食品是食品的一个种类，具有一般食品的共性，能调节人体的机能，适用于特定人群食用”的态度是？[单选题]*

| ①非常赞同 |
| --- |
| ②赞同 |
| ③一般 |
| ④不赞同 |
| ⑤非常不赞同 |

32.小明近期体检发现他骨密度指数偏低，提示高骨质疏松风险，有朋友担心他身体状况，向他推荐了钙片、复合维生素、鱼油、益生菌等一系列保健食品，您觉得小明应该如何选择？[单选题]*

| ①相信朋友的推荐，立刻全部购买 |
| --- |
| ②不相信保健食品，不接受朋友的推荐 |
| ③比较同类产品，优选性价比高的购买 |
| ④结合专业人士建议、产品标志、包装标识和适宜人群等因素综合选购适合自己的 |
| ⑤不知道 |

33.您对“在食物清洗、切配、储藏的整个过程中，生熟都应分开”的态度是？[单选题]*

| ①非常赞同 |
| --- |
| ②赞同 |
| ③一般 |
| ④不赞同 |
| ⑤非常不赞同 |

34.您或者您家做饭的人在生活中总会用不同的器具来盛装生食和熟食吗？[单选题]*

| ①完全不会 |
| --- |
| ②极少会 |
| ③有时会 |
| ④经常会 |
| ⑤总是会 |

35.从冰箱里把还可以食用的剩菜拿出来重新吃之前，您会？[单选题]*

| ①不影响口感的话直接吃 |
| --- |
| ②放在室温下过一会再吃 |
| ③用锅或者微波炉稍微热一下再吃 |
| ④彻底加热（加热到100℃或微波炉高火2分钟）后再吃 |
| ⑤不知道 |

36.您对“热带水果（如香蕉）也可以放在冰箱储藏”的态度是？[单选题]*

| ①非常赞同 |
| --- |
| ②赞同 |
| ③一般 |
| ④不赞同 |
| ⑤非常不赞同 |

37.您对“在冰箱中，应该熟食放在上层，生食放在下层”的态度是？[单选题]*

| ①非常赞同 |
| --- |
| ②赞同 |
| ③一般 |
| ④不赞同 |
| ⑤非常不赞同 |

38.如果你中午炒了一盘红烧肉，剩了一大盘，你一般会如何处理？[单选题]*

| ①直接倒掉 |
| --- |
| ②用保鲜膜或保鲜盒装好放在桌子上 |
| ③直接放进冰箱（无保鲜膜或保鲜盒包裹） |
| ④用保鲜膜或保鲜盒装好，放进冰箱 |
| ⑤不知道（不在家里吃或者不负责处理饭菜） |

39.您在点外卖的时候，会考虑查看外卖店铺的卫生相关信息吗？[单选题]*

| ①完全不会 |
| --- |
| ②极少会 |
| ③有时会 |
| ④经常会 |
| ⑤总是会 |

40.您在外就餐选择就餐点时，会优先考虑就餐点的卫生情况吗？[单选题]*

| ①完全不会 |
| --- |
| ②极少会 |
| ③有时会 |
| ④经常会 |
| ⑤总是会 |

41.您如果在外就餐，会选择哪个卫生等级以上的餐馆？[单选题]*

| ①A级 |
| --- |
| ②B级 |
| ③C级 |
| ④不会注意等级 |
| ⑤不知道 |

42.您平时会定期监测自己的体重情况吗？[单选题]*

| ①完全不会 |
| --- |
| ②极少会 |
| ③有时会 |
| ④经常会 |
| ⑤总是会 |

43.BMI（体质指数）是用来评价人体营养状况的常用方法，公式：BMI(kg/m2)=体重(kg)/身高(m)2，下图为BMI的中国标准范围表。小明的体重为72公斤，身高为170厘米，请您评价一下他目前的营养状况。


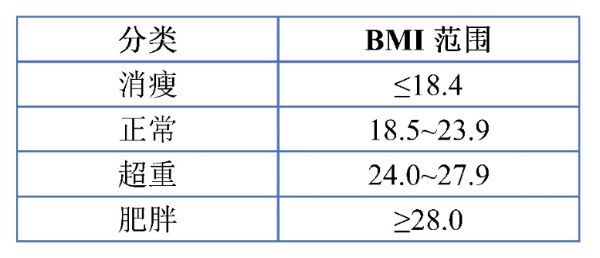


[单选题]*

| ①消瘦 |
| --- |
| ②正常 |
| ③超重 |
| ④肥胖 |
| ⑤不知道 |
